# Are Functional and Activity Limitations Becoming More Prevalent among 55 to 69-Year-Olds in the United States?

**DOI:** 10.1371/journal.pone.0164565

**Published:** 2016-10-26

**Authors:** HwaJung Choi, Robert F. Schoeni, Linda G. Martin

**Affiliations:** 1 Department of Internal Medicine, University of Michigan, Ann Arbor, MI, United States of America; 2 Department of Economics, University of Michigan, Ann Arbor, MI, United States of America; 3 G. Ford School of Public Policy, University of Michigan, Ann Arbor, MI, United States of America; 4 Survey Research Center, University of Michigan, Ann Arbor, MI, United States of America; 5 RAND Corporation, Arlington, VA, United States of America; Beijing Key Laboratory of Diabetes Prevention and Research, CHINA

## Abstract

**Objectives:**

This study examines changes in functional and activity limitations 1998–2012 for individuals 55–69.

**Methods:**

Logistic models are used to estimate trends in limitations in vision, hearing, physical and cognitive functioning, IADLs, and ADLs. Additional models assess the extent to which trends are associated with and differ by education, smoking, and BMI.

**Results:**

Changes in prevalence of limitations in vision, hearing, cognitive functioning, and ADLs are not statistically significant. Limitations in physical functioning declined by 0.37% per year. IADL limitations increased by 1.33% per year, but most of the increase occurred between 2008 and 2010/2012, and are associated with economic hardship during the Great Recession. Increases in education are especially beneficially associated with trends in limitations, but reductions in smoking also appear to be advantageous for some outcomes. Increases in BMI are associated with trends in physical functioning, IADL, and ADL limitation.

**Discussion:**

For Americans 55–69, functional and activity limitations were largely unchanged 1998–2012. Our results suggest that if educational attainment had not increased, most functional and activity limitations potentially could have worsened substantially. Future change in educational attainment is not expected to be so positive. Continued monitoring of trends in activity limitations might well include greater focus on the explanatory roles of environmental factors, including economic circumstances.

## Introduction

The rapid decline in the prevalence of functional and activity limitations among the 65 and over population in the 1980s and 1990s is well documented [1]. However, there is evidence from several national surveys that these improvements may have plateaued in the early 2000s [2, 3]. Moreover, there is growing concern that the prevalence of functional and activity limitations at pre-retirement ages may even have increased [4–8], a troubling trend not only for these cohorts but for the future of health care and related spending.

Attention of the research and policy communities also has focused on the possibility of increasing differentials across subgroups in health, more broadly defined. A recent National Research Council report (2015) [9] and Bound and colleagues (2015) [10] highlighted growing socioeconomic differentials in life expectancy in the United States. The review of literature by Hummer and Hernandez (2013) [11] suggests that health behaviors such as smoking, nutrition, and physical activity account for about 30 percent of the educational difference in survival. Analyzing trends in the prevalence of activity limitations, Schoeni and colleagues (2005, 2009) [12, 13] found increasing disparities by education and income for the 70 and over population from 1982 to 2002.

Understanding what factors might account for the trends in limitations has also been a focus of research on the well-being of the older population. Schoeni and colleagues (2008)[14] in their analysis and broad review of the literature found that increased educational attainment and medical advances in cardiovascular disease, cataracts, and arthritis played important roles.

This study focuses on the U.S. population ages 55 to 69 and uses national data from 1998 to 2012 from the Health and Retirement Study (HRS) to address three questions. The first objective is to update trends in a comprehensive set of measures of functional and activity limitations. Most trend studies of the pre-retirement population have relied on data from the National Health Interview Survey (NHIS), and the most recent detailed published analysis using them extends only to 2010 [6]. Trend analysis by Freedman and colleagues using HRS data ends in 2008 [2]. Furthermore, none of these studies have examined physical functioning, cognitive functioning, and activity limitations together to provide a more complete understanding of the experiences of this younger age group.

The second objective is to assess the association of trends in education, smoking experience, and body mass index with trends in functional and activity limitations. These three factors are the focus because they are strongly associated with health in the cross section, and, as shown below, their distributions changed substantially over the study period for this cohort. Martin and Schoeni (2014) [6] in their analysis of 1997–2010 NHIS data for the population ages 40 to 64 years found that trends in body mass index potentially could account for the upward trend in physical functional limitations and some activity limitations. Increased education and reduced smoking over time were associated with beneficial trends in activity limitations for males. The HRS data provide an opportunity for replication of this analysis for a slightly older population.

The third objective is to test for subgroup differentials in trends in functional and activity limitations by education, smoking experience, and body mass index. To our knowledge, no one has investigated such trends in disparities for Americans in their pre-retirement years.

Monitoring these trends and understanding their causes during the pre-retirement years is important because functional and activity limitations are key indicators of health and well-being. The time period 1998–2012 was marked by increases in inequality [15] and the tremendous economic turmoil of the Great Recession, prompting a need to determine whether disparities in functional and activity limitations worsened.

## Methods

### Data

We used data for respondents ages 55 to 69 years from the 1998–2012 HRS, a nationally representative longitudinal survey. Telephone or in-person interviews with HRS participants are conducted every 2 years. Cross-sectional weights for each survey wave allowed us to analyze the data as a time series of cross sections of a nationally representative sample. Because the weights for institutionalized respondents were not available for 1998 and 2012 at the time of preparing this manuscript, we included only community-dwelling respondents for our primary analysis sample. However, based on 2000–2010 data, we conducted sensitivity analyses of general trends that included the institutionalized population, and not surprisingly, given the small size of the institutionalized population for the 55–69 group (0.4% in 2010), the results (described below) did not differ substantially from those of our main analysis. Similarly, we stratified our trend analyses by gender, but found very few substantive differences (exceptions are described below) and therefore report estimates for men and women combined.

The total number of person-year observations in our sample for those ages 55 to 69 for the 15-year period is 72,470, ranging from 7,771 in 2008 to 10,467 in 1998. The size of the analysis sample differed across limitation outcomes, as indicated in Table 1, because the rate of missing data differed across outcomes. The analysis of cognitive function trends had the smallest sample size (68,189) of all outcomes because we rely solely on self-responses and did not include proxy responses. For the other limitation measures, proxy responses were included, and altogether item non-response ranged from 0.05% (hearing) to 5.25% (instrumental activities of daily living).

**Table 1:** Weighted unadjusted prevalence of functional limitations, activity limitations, and covariates for ages 55–69, living in the community, 1998–2012, percent unless otherwise indicated.

	1998	2000	2002	2004	2006	2008	2010	2012	Average: 1998–2012	Unweighted N
Functional limitations										
Vision: poor or legally blind	3.77	3.89	3.41	3.78	3.61	3.50	3.88	4.37	3.78	72,407
Hearing: poor	3.26	2.82	3.13	3.29	3.59	3.25	3.17	3.69	3.27	72,431
Cognition: CIND or demented (self-reports only)	11.34	11.82	10.72	11.48	11.92	12.09	11.96	12.79	11.77	68,189
Physical functioning: Any of 9 limitations	56.85	57.16	58.41	59.45	59.87	58.49	57.29	55.72	57.91	71,147
Activity limitations										
Any of 5 IADLs	8.93	9.49	9.38	9.56	10.12	9.62	11.24	11.22	9.95	68,667
Some difficulty with using a telephone	1.89	2.04	1.90	1.56	1.74	1.68	2.24	2.19	1.91	72,184
Some difficulty with handling money	2.68	2.84	2.96	3.07	3.29	3.17	4.21	4.11	3.29	70,648
Some difficulty with taking medication	1.59	1.74	1.78	1.87	2.07	2.28	2.43	2.23	2.00	72,282
Some difficulty with shopping for groceries	5.45	5.70	5.55	5.26	6.06	5.22	6.58	6.43	5.78	71,039
Some difficulty with preparing meals	3.12	3.29	3.28	3.21	3.38	2.88	3.71	3.72	3.32	70,279
Any of 6 ADLs	12.01	12.27	11.95	12.40	13.52	11.89	13.51	12.50	12.51	72,337
Covariates (unweighted N)	10,467	9,818	9,289	8,912	8,327	7,771	8,917	8,969	-	72,470
Mean age	61.31	61.37	61.18	61.01	61.02	61.14	61.15	61.27		
Female	53.01	52.82	52.92	52.24	52.45	52.24	52.61	52.58		
Proxy respondent	7.14	7.78	8.34	6.93	4.48	3.94	3.63	3.01		
In-person interview	20.06	6.92	5.86	66.14	49.72	44.90	59.06	48.48		
Years of education:										
< High school or GED	22.42	20.43	17.90	16.37	14.97	13.31	11.85	11.10		
High school or GED	37.08	36.83	36.50	35.21	34.00	32.77	30.41	30.35		
Some college	20.13	21.12	22.53	23.57	24.53	26.04	27.05	28.07		
> = Bachelor's degree	20.37	21.62	23.07	24.85	26.50	27.88	30.70	30.48		
Smoking:										
Never	37.25	37.39	38.30	39.56	40.68	42.15	43.44	43.73		
Former	42.39	43.38	43.09	41.26	41.15	40.45	39.49	39.10		
Current	20.37	19.23	18.61	19.17	18.17	17.40	17.07	17.17		
BMI:										
<18.5	1.26	0.99	0.98	0.99	0.89	0.96	1.05	1.09		
18.5–24.99	31.76	29.93	28.62	28.37	25.08	23.82	24.56	24.75		
25–29.99	41.03	41.11	40.11	38.73	39.14	38.40	36.99	36.53		
30–34.99	18.18	18.93	19.63	20.68	22.18	22.45	22.59	22.39		
35–39.99	5.28	5.95	7.20	7.09	7.88	9.12	9.14	9.68		
> = 40	2.49	3.08	3.47	4.13	4.83	5.26	5.69	5.56		
Taken less medication because of cost (last 2 years)	5.83	7.20	8.02	10.07	10.45	10.04	13.06	12.27		
Ever not enough money for food you need (last 2 years)	8.66	5.06	5.15	6.48	6.73	6.99	9.44	10.61		

### Measures of Limitations

In selecting measures of limitations, we relied on the conceptual framework of the disablement process, as outlined by Verbrugge and Jette (1994) [16]. In that process, a disease (such as arthritis) may result in an impairment (such as joint stiffness). In turn, the impairment may lead to a functional limitation including physical (such as difficulty climbing stairs), cognitive, and sensory limitations. A functional limitation, which represents the underlying capacity of an individual, may then result in a disability. The concept of disability is often operationalized as a limitation in an activity. Activities may include household tasks (e.g., shopping and preparing meals), which are often referred to as instrumental activities of daily living (IADLs), and personal care tasks (e.g., bathing and dressing), which are called activities of daily living (ADLs). These IADL and ADL measures of disability represent a gap between underlying capacity and the demands of a specific activity as it is carried out in a particular built and social environment.

In this paper, we assess trends in vision, hearing, cognitive, and physical functioning limitations, as well as limitations in IADLs and ADLs. For vision limitation, we use a binary indicator of self-reported poor vision (using glasses or corrective lens as usual) or legal blindness. Hearing limitation is indicated by reported poor hearing (using a hearing aid as usual).

Our indicator of cognitive limitation is based on a summary measure developed by Langa and Weir [17, 18]. They use the items from the modified Telephone Interview for Cognitive Status (TICS), excluding items having to do with orientation since they are not asked of younger respondents in the HRS [19]. The total score of cognitive functioning ranges from 0 to 27 points and represents the sum of: immediate word recall (0–10 points); delayed word recall (0–10 points); serial 7s (0–5 points); and backwards counting from 20 (0–2 points). A greater number of points reflects better cognitive functioning. In the Langa-Weir specification, a total score of 0–6 points is labeled as demented, 7–11 as cognitively impaired but not demented (CIND), and 12–27 as normal. We created an indicator of poor cognition by assigning 1 for demented or CIND, and 0 otherwise.

Physical functional limitation is a dichotomous variable indicating self-reported difficulty with at least one of nine functions: climbing a flight of stairs without resting; getting up from a chair; picking up a dime; reaching/extending arms up; pushing or pulling large objects; sitting for 2 hours; lifting and carrying 10 pounds; stooping, kneeling, and crouching; and walking several blocks.

The measure of IADL limitation is an indicator of having difficulty because of a health or memory problem with at least one of five activities: making phone calls, handling money, taking medication, shopping for groceries, and preparing a hot meal. For ADL limitation, we created an indicator of having difficulty with at least one of six items: walking across a room, dressing, bathing, eating, transferring in/out bed, and toileting. Space constraints preclude analysis of trends of each of the specific IADL and ADL limitations, however, as noted later, because of a nonlinearity in the time trend of the summary indicator of IADL limitation, we do explore trends in limitations in specific IADLs.

### Explanatory Factors

In all our models, we controlled for age (with a quadratic specification), gender, an indicator of response by a proxy respondent, and an indicator of in-person interview (as opposed to by phone). We also explored additional factors with strong cross-sectional relations with limitations and whose prevalence changed over time, namely, years of education (<high school or GED, high school or GED, some college, bachelor’s degree or higher), smoking (never smoked, former smoker, current smoker); and body mass index (BMI). BMI was calculated using self-reported weight and height and categorized in the following groups: <18.5, 18.5–25, 25–30, 30–35, 35–40, and > = 40. In preliminary models, we also included measures of race/ethnicity, being foreign born, and marital status (in S1 Table). However, the trend estimates did not change substantially with their inclusion, suggesting that these variables did not have the potential to explain trends in limitations.

We found that IADL limitations were unusually high in 2010 and 2012, right after the onset of the Great Recession. To assess whether economic hardship brought on by the Great Recession could account for this large, discrete increase in IADL limitations, we estimated a model for 1998 to 2012 that included indicators of whether over the previous two years respondents had taken less prescription medication because of cost and whether respondents did not have enough money for food in the previous two years.

### Statistical Analysis

We used multivariate logistic regressions to estimate change over time in each of the measures of functional and activity limitation. Our basic model (Model 1 in Tables 2 and 3) adjusted for age, gender, proxy status, and survey mode to assess the trend. To estimate the trend, we included in the models a continuous variable of survey year that took the value of the calendar year in which the particular data point was collected, for example, 1998 for data collected in survey year 1998. For each model, we estimated the adjusted annual percent change of the limitation outcome using adjusted risk ratios (ARR), as suggested by Norton, Miller, and Kleinman (2001) [20]. In our application, the ARR is the ratio of the predicted probability of the outcome for 2012 to the predicted probability of the outcome for 1998. These predicted probabilities are calculated using the estimated parameters from models based on the data for all survey waves from 1998 to 2012. The estimated annual percentage change is 100 * ln(ARR_2012 vs. 1998_) /14. We used this approach rather than simply calculating the rate of change as 100*(odds ratio for the survey year variable minus 1), since for outcomes whose risks are high, the odds ratio may diverge from the ARR [20].

**Table 2:** Adjusted annual percent change in functional limitations and activity limitations based on multivariate logistic regressions with various controls, 1998–2012 (95% confidence intervals are in parentheses).

Outcome	Model 1	Model 2	Model 3	Model 4
Functional limitations				
Vision: Poor or legally blind (N = 72,407)	0.20%	2.35%	0.55%	-0.11%
	(-1.06 1.45)	(1.19 3.51)	(-0.69 1.78)	(-1.35 1.13)
Hearing: Poor (N = 72,431)	0.89%	1.82%	1.14%	0.56%
	(-0.36 2.13)	(0.59 3.06)	(-0.12 2.40)	(-0.68 1.80)
Cognition: CIND or demented (self-reports only; N = 68,189)	0.20%	2.79%	0.36%	0.00%
	(-0.62 1.03)	(2.20 3.38)	(-0.45 1.18)	(-0.83 0.82)
Physical functioning: any of 9 limitations (N = 71,147)	-0.37%	0.10%	-0.27%	-0.72%
	(-0.63 -0.10)	(-0.14 0.34)	(-0.53 -0.01)	(-0.95 -0.49)
Activity limitations				
Any of 5 IADLs (N = 68,667)	1.33%	2.76%	1.61%	0.69%
	(0.59 2.08)	(2.07 3.45)	(0.86 2.36)	(-0.02 1.40)
Any of 6 ADLs (N = 72,337)	0.02%	1.34%	0.22%	-0.88%
	(-0.77 0.80)	(0.62 2.07)	(-0.55 1.01)	(-1.60 -0.15)
Controls				
Age, gender, proxy, mode	x	x	x	x
Education		x		
Smoking			x	
Obesity				x

**Table 3:** Adjusted annual percent change in limitations in individual IADLs, controlling for economic hardship, 1998–2012 (95% confidence intervals are in parentheses).

Outcome	Model 1	Model 2
Any of 5 IADLs	1.33%	0.29%
	(0.59 2.08)	(-0.42 0.99)
Some difficulty with using a telephone	1.44%	0.40%
	(-0.38 3.25)	(-1.33 2.13)
Some difficulty with handling money	3.28%	2.13%
	(2.18 4.37)	(1.10 3.17)
Some difficulty with taking medication	3.17%	1.93%
	(1.62 4.71)	(0.34 3.53)
Some difficulty with shopping for groceries	0.99%	-0.09%
	(0.06 1.92)	(-0.96 0.78)
Some difficulty with preparing meals	1.26%	0.14%
	(0.18 2.34)	(-0.87 1.16)
Controls		
Age, gender, proxy, mode	x	x
Economic hardship: Taken less medication because not enough money		x
Economic hardship: Not enough money for food		x

To assess the extent to which education, smoking, and obesity could possibly account for the trends we found in functioning and activity limitations, we added each of these factors to models with the basic covariates (Model 2, Model 3, and Model 4, respectively, in Table 2). Finally, to investigate whether economic hardship variables influence responses to IADL questions and hence explain the unusually large increase in IADL limitations in 2010, as described above, we included the two economic hardship measures in addition to the basic covariates (Model 2 in Table 3).

In our analysis of disparities in trends by subgroups, we focused on the same three factors highlighted in the analysis seeking to account for the trends of each limitation outcome, namely, education, smoking, and obesity. For example, in the education disparities model, in addition to the basic covariates and the education group variables, we included interactions between survey year and education group. Using the model results, we calculated the ARR and implied annual percent change for each education group and type of limitation. We tested differences in trends across education groups using the significance of the interaction terms in models with different education reference groups. The same approach was used for the smoking disparities and obesity disparities models.

In all three parts of the analysis (trends, explanations, disparities), when a particular covariate was included, we also controlled for item non-response for that covariate by using an indicator of missingness of that covariate in the model. In that way, we avoided dropping observations with missing values for the covariates. Rates of item non-response in covariates ranged from 0.0% for sex to 1.63% for BMI. In all analyses, which we conducted using STATA 14 software, we took into account the complex survey design of the HRS. STATA codes created to obtain (adjusted) annual percent change of the limitation outcomes are available upon request.

## Results

Table 1 reports, for each year, the weighted prevalence of each functional and activity limitation as well as of the explanatory factors. The prevalence estimates are not adjusted for any factor such as age or gender, and they are reported here to illustrate the magnitude of the prevalence of each outcome and how the prevalence changes from year to year

Problems with vision and hearing are relatively uncommon over the 14-year period from 1998 to 2012, with only 3.78% reporting being legally blind or having poor vision, and 3.27% reporting poor hearing, on average during these years. Based on self-reports only, 11.77% of the population ages 55 to 69 years is categorized as having cognitive impairment but not being demented or being demented (CIND). For the full sample including proxies, 57.91% reported limitation in physical function, on average 1998 to 2012.

Difficulty with at least one IADL is reported by 8.93 to 11.23% of those ages 55 to 69, depending on the year. Among the individual IADLs, difficulty shopping for groceries is the most common with over 5% reporting difficulty. On average between 1998 and 2012, 12.51% reported difficulty with at least one of the six ADLs.

The average age of our analysis sample is just over 61 years and changes very little between 1998 and 2012. The gender composition of the population also remains fairly steady, with just under 53% women. Use of proxy respondents was more common in the first few years than the last few; for example, the share of interviews completed by proxies was 8.34% in 2002 and 3.01% in 2012. The decline in proxy responses has been linked to the increase in the share of interviews conducted face-to-face, as HRS shifted to collecting biomarkers and physical measures, beginning with a random subsample of respondents in 2004 [21, 22]. Also, although prior to 2006 telephone was the primary mode of interviewing for respondents under age 80, exceptions were made for first interviews with members of birth cohorts added in 1998 and 2004 [23].

Years of completed education were substantially higher in 2012 than 1998. In 1998 22.42% of individuals had less than a high school degree or GED, but by 2012 this share had declined to 11.10%. The proportion with at least a bachelor’s degree increased from 20.37 to 30.48%. Smoking became less common: the proportion currently smoking fell from 20.37% in 1998 to 17.17% in 2012. Over the same period, BMI increased: the percentage in the top two groups combined (BMI> = 35) increased from 7.77 to 15.24%.

Finally in Table 1 are the percentages of respondents reporting that over the past two years, they took less prescription medication than was prescribed because of cost and they did not have enough money to buy the food they needed. Both increased substantially between 2008 and 2010.

### Basic Models

The first column of Table 2 presents the estimated annual percentage changes in functional and activity limitations from the basic models (Model 1). No statistically significant trends were observed for vision, hearing, cognitive, or any ADL limitation. A small statistically significant decline of 0.37% per year is estimated for difficulty with any of the 9 physical functions. However, for difficulty with any of 5 IADLs, the estimated change is positive, statistically significant, and large: 1.33% per year.

Several additional analyses were conducted to test the robustness of these findings to various factors. First, all models were estimated separately for men and for women to determine whether the findings differ across these two groups (in S2 Table). The only estimates that were statistically significant in the basic model were for women: IADL (1.6% per year) and physical functioning (-0.5% per year).

Second, we also found that including in the analysis the less than 1% of the population in this age range that is institutionalized does not influence the conclusions substantially (S3 Table, which is based on 2000–2010 data). Third, nonlinearities in trends were assessed through graphical inspection, and the only substantial nonlinearity detected was for IADLs between 2008 and 2010/2012, a finding that is discussed below in the context of the results presented in Table 3.

### Possible Explanations of Trends

Model 2 (in Table 2) in which education is added to the basic covariates yields less favorable trends in all limitation outcomes. This specification suggests that if educational attainment had not increased over time for the 55–69 age group (essentially controlled for in Model 2), limitation in vision might have increased by an estimated 2.35% per year, hearing by 1.82% per year, cognition by 2.79% per year, IADLs by 2.76% per year, and ADLs by 1.34% per year. The estimated decline in difficulty with the 9 physical functions from Model 1 becomes statistically insignificant in Model 2.

Controlling for smoking behavior (Model 3 in Table 2) has relatively modest effects on estimates of trends in the outcomes. For vision, hearing, cognition, and any ADL, both the basic model and the model controlling for smoking imply no statistically significant trend. For physical functioning, the statistically significant decline estimated in the basic model (-0.37%) remains statistically significant but becomes somewhat smaller (-0.27%) when smoking is controlled. Similarly for any IADL, the statistically significant increase estimated in the basic model (1.33%) remains statistically significant but becomes somewhat larger (1.61%) once smoking is controlled.

Including BMI (Model 4 in Table 2), the estimated upward trend in IADL limitation is no longer significantly different from zero. Furthermore, the estimated annual change in ADL limitation shifts from insignificance in Model 1 to significantly downward in Model 4 (-0.88%), again suggesting a possible deleterious effect of the change in BMI distribution over time.

Visual inspection of the year-to-year change in prevalence of difficulty with any of the 5 IADLs reveals substantial nonlinearities (Table 1). In particular, much of the increase in the prevalence arises between 2008 and 2010. During the ten-year period 1998 and 2008, the prevalence rose by 0.69 percentage points, but in the two-year period 2008 to 2010 the prevalence rose by a relatively large 1.62 percentage points to 11.24% and stayed at this higher level in 2012. Inspection of the change in prevalence of each of the five individual IADL items (Table 1) also indicates unusually large increases for several items between 2008 and 2010. Although there were increases late in our study period in the raw prevalence of sensory and cognitive functional limitations, which underlie activity limitations in our framework, these increases were not nearly as dramatic as the ones for IADLs and were not statistically significant in our basic models (Table 2). Moreover, we found a decline in physical functional limitations. Accordingly, we were led to consider environmental factors to explain the large increase in IADL limitations.

The timing of the increase is important because the effects of the Great Recession on poverty were large between 2008 and 2010–12; after staying at roughly 13% between 2003–2008, the poverty rate jumped to 15.1% in 2010 and remained high at 15.0% in 2012 [24]. This general pattern is reflected in the trends for the indicators of material hardship reported in the last two lines of Table 1: taking less prescription medication because of cost and not having enough money for food. Respondents were instructed to report difficulty with the IADL items if it was because of a health or memory problem. However, in the context of a lengthy interview, this qualification may not always have been retained by the respondents. For example, it could be that a respondent would report difficulty shopping for groceries because of not having enough money.

To investigate whether changes in material hardship, likely induced by the Great Recession, could potentially account for the rise in IADL limitation, we added the two hardship indicators to the basic model for IADL trend, as well as estimating similar models for each of the five individual IADL items. As shown in Table 3, controlling for these two indicators results in the estimated annual change in the prevalence of difficulty with any of 5 IADLs decreasing from a statistically significant 1.33% to a much smaller and insignificant 0.29%. The individual IADL items follow a fairly similar pattern, with the estimated trend becoming substantially smaller. Most notably, the estimated annual percentage changes for two of the individual items—difficulty shopping for groceries and difficulty preparing meals—are no longer significantly different from zero after controlling for economic hardship.

### Disparities in Trends

Evidence on differential trends across education groups is mixed (Table 4). For vision, cognition, and ADL limitations, across all four education groups the prevalence of these limitations is generally significantly increasing; the exceptions are vision among individuals in the highest and lowest groups and ADLs for the highest. The differences in estimated trends across the education groups (right side of Table 4) are not statistically significant for any of these three outcomes.

**Table 4:** Adjusted annual percent change in functional and activity limitations based on multivariate logistic regressions with various controls and interactions of education and trend, 1998–2012 (95% confidence intervals are in parentheses).

					p-value of adjusted risk ratio (2012 vs. 1998) for difference in trend across groups					
Outcome	<HS/GED	HS or GED	Some college	BA or more	<HS/GED = HS/ GED	<HS/GED = Some college	<HS/GED = BA or more	HS/GED = Some college	HS/GED = BA or more	Some college = BA or more
Functional limitations										
Vision: Poor or legally blind	1.40%	2.43%	4.21%	3.17%	0.478	0.148	0.443	0.277	0.740	0.684
	(-0.28 3.08)	(0.63 4.24)	(1.22 7.21)	(-0.74 7.07)						
Hearing: Poor	-0.84%	1.97%	4.12%	2.58%	0.116	0.016	0.053	0.187	0.750	0.394
	(-3.39 1.71)	(-0.16 4.11)	(1.62 6.62)	(-0.20 5.37)						
Cognition: CIND or demented (self-reports only)	2.22%	2.82%	4.57%	3.05%	0.665	0.260	0.789	0.146	0.961	0.382
	(1.43 3.01)	(1.84 3.81)	(2.77 6.37)	(0.31 5.79)						
Physical functioning: Any of 9 limitations	0.22%	0.46%	0.05%	-0.66%	0.562	0.436	0.012	0.103	0.001	0.086
	(-0.10 0.55)	(0.10 0.82)	(-0.45 0.54)	(-1.20 -0.11)						
Activity limitations										
Any of 5 IADLs	1.54%	3.78%	3.42%	1.89%	0.045	0.126	0.977	0.642	0.154	0.189
	(0.20 2.89)	(2.53 5.03)	(2.04 4.81)	(-0.30 4.08)						
Any of 6 ADLs	1.53%	1.36%	1.63%	0.36%	0.698	0.862	0.216	0.799	0.334	0.196
	(0.38 2.67)	(0.06 2.66)	(0.35 2.90)	(-1.58 2.31)						
Controls										
Age, gender, proxy, mode	x	x	x	X						

Similarly for IADL limitation, all groups but the most educated experienced significant increases in limitations; however, the only statistically significant differences in estimated trends were high school graduates/GED holders versus those with a lower level of education, with the least educated group experiencing a smaller increase in the prevalence of IADL limitation (1.54% vs. 3.78%).

Only for those in the some college group is there a significant increase in hearing limitation. Moreover, the hearing trend for individuals with some college was significantly higher than the hearing trend for individuals with less than high school or a GED.

Trends in difficulty in physical functioning show just the opposite educational differentials. That is, it is only for the most educated group that the prevalence of such a limitation declined significantly (-0.66% per year), whereas the trend for those with a high school diploma or GED was significantly positive (0.46% per year). Moreover, the difference in trends across education groups is statistically significant between those with a BA or more and the two least educated groups (<HS/GED, HS or GED).

For current smokers (Table 5), the estimated annual percent change for all outcomes except poor hearing was positive, that is, unfavorable, and statistically significant: 1.93% for vision, 1.82% for cognition, 0.47% for physical functioning, 3.07% for IADLs, and 1.70% for ADLs. Trends for current smokers were much less favorable than for those who had never smoked for all outcomes except poor hearing. Former smokers had more favorable trends than current smokers for four of the six types of limitations: cognition, physical functioning, IADLs, and ADLs. Differences in trends for never versus former smokers were not statistically significant.

**Table 5:** Adjusted annual percent change in functional and activity limitations based on multivariate logistic regressions with various controls and interactions of smoking and trend, 1998–2012 (95% confidence intervals are in parentheses).

				p-value of adjusted risk ratio (2012 vs. 1998) for difference in trend across groups		
Outcome	Current smoker	Former smoker	Never smoked	Current = Never	Current = Former	Former = Never
Functional limitations						
Vision: Poor or legally blind	1.93%	0.72%	-1.08%	0.028	0.271	0.158
	(0.11 3.76)	(-1.03 2.47)	(-3.13 0.97)			
Hearing: Poor	1.08%	1.77%	0.20%	0.660	0.596	0.354
	(-0.91 3.07)	(-0.19 3.73)	(-2.48 2.89)			
Cognition: CIND or demented (self-reports only)	1.82%	0.13%	-0.49%	0.002	0.011	0.388
	(0.67 2.97)	(-0.88 1.14)	(-1.74 0.77)			
Physical functioning: Any of 9 limitations	0.47%	-0.44%	-0.49%	0.000	0.000	0.880
	(0.11 0.82)	(-0.79 -0.10)	(-0.83 -0.14)			
Activity limitations						
Any of 5 IADLs	3.07%	1.29%	0.71%	0.003	0.013	0.471
	(1.83 4.32)	(0.28 2.30)	(-0.57 2.00)			
Any of 6 ADLs	1.70%	-0.22%	-0.31%	0.004	0.011	0.906
	(0.58 2.82)	(-1.29 0.84)	(-1.43 0.82)			
Controls						
Age, gender, proxy, mode	x	x	x			

Models stratified by BMI (<30, 30–34, > = 35) were also estimated, but the results are not reported here, because at the 0.05 level there were no statistically significant differences in trends across the BMI groups.

## Discussion

Overall, the prevalence of limitations in vision, hearing, cognition, and ADLs did not change significantly between 1998 and 2012 for adults ages 55–69. Physical functioning limitation declined by 0.37% per year. The lack of change in ADL limitation and the only modest decline in physical functioning limitation for this age group stand in contrast to the substantial downward trends in such limitations that have been found for older age groups of Americans for the 1990s [1, 25]. However, the results are consistent with the flat trend in ADL limitations for the older population that has been found in analysis of data from 2000 to 2008 from multiple national surveys [2].

The prevalence of IADL limitation for those ages 55 to 69 years increased by 1.33% per year—again in contrast to the older population for whom IADL limitation declined in preceding decades [1] and did not change from 2000 to 2008 [2]. However, almost all of the increase in IADL limitation for the younger group occurred between 2008 and 2010/2012, the period when there was substantial economic turbulence associated with the Great Recession. We find that adjusting for two measures of economic hardship—taking less medication because of not having enough money, and not having enough money for food—eliminates the estimated increase, implying no change over this period.

This finding suggests that additional research is needed to assess the sensitivity to economic conditions of responses to survey questions about limitations in IADLs. It is quite possible that instead of capturing changes in limitations in activities such as handling money and preparing meals that are related to health challenges, surveys are capturing changes in limitations in these activities due to the fact that the respondents experience changes in financial wherewithal to purchase items such as groceries. Furthermore, these findings suggest that research whose objective is to estimate long-term trends in activity limitations—and IADL limitations in particular—need to be mindful that such measures may change rapidly because of changes in economic, social, and built environments in which these activities are carried out. When possible, analyses should examine data for discontinuities and nonlinearities over time and not simply focus on the endpoints. Accordingly, it will be important to update this analysis of trends when data collected after 2012 are available to determine if the prevalence of IADL limitations declined as the economic situations of Americans recovered from the Great Recession.

Educational attainment is one of the strongest correlates of health and disability, and education levels improved tremendously over the study period. Among individuals 55–69 years old in 1998, 22% did not have a GED or high school diploma, but by 2012, only 11% of individuals 55–69 had this level of education. In comparison to our basic models, our models that control for education, and thus eliminate its potentially beneficial influence, result in worse trends in the estimated prevalence of functional and activity limitations. In the models with education, the estimated annual percent increases are substantial for limitations in vision (2.35%), hearing (1.82%), cognition (2.79%), IADLs (2.76%), and ADLs (1.34%), and the declines in physical functioning limitation are eliminated.

Future trends in educational attainment will likely not be so dramatic as in past decades. We calculated simple projections of educational attainment that do not adjust for educational differentials in mortality, using data for people ages 30–69 in 2012 from the Current Population Survey (in S4 Table). These projections indicate that among people 55–69 years old, the proportions in the lowest education group and in the some college group will change little from 2012 to 2037 (roughly 11 and 27% in 2012, and 11 and 28% in 2037, respectively). The proportion with a high school education is projected to decline from 31 to 27% and the proportion with a bachelor’s degree or more to increase from 31 to 35% over the same period. Of course, the implications for health of belonging to a particular education group may well change over time [10].

Some of the disparities in trends by education group that we found reflected a reduction in disparities. For example, those with some college had greater increases in difficulty hearing than those with less education. One possibility is there were differential changes in environmental exposures to and protections from excessive noise. Nevertheless, the prevalence of hearing difficulty remained highest for the least educated group throughout the study period (in S5 Table).

The increase in IADL limitation among the least educated also is less than that for those with a high school education. We have demonstrated that trends in economic hardship are associated with trends in IADL limitation. Those with less than a high school education may not have experienced as great an increase in hardship, either because they were already living with limited means before the Great Recession or because they were relying on the social safety net. As with hearing, the prevalence of IADL difficulty remained highest for the least educated group throughout the study period (in S5 Table).

Changes in smoking were not as strongly associated as changes in education with the observed trends in limitations, although their effects appear to be beneficial. Moreover, trends in all outcomes except hearing were more favorable for individuals who had never smoked than those who are current smokers. Our models controlling for BMI suggest that if the BMI distribution had not worsened, the prevalence of ADL limitation might have declined and the increase in the prevalence of IADL limitation might have been dampened. But differential trends by BMI group were not statistically significant at the 0.05% level. That said, the effects of quitting smoking or becoming obese may occur with a lag, so it could be that these factors will play larger roles in trends in limitations in the future.

Much more is to be learned about the roles of the three factors highlighted here in trends in limitations. Also warranted is investigation of other variables potentially associated with the trends, such as medical interventions and, for activity limitations, changes in the use of assistive technology and changes in the built environment. Monitoring these trends and understanding their causes during the pre-retirement years may provide useful insight into future prospects for living independently well into old age.

## Supporting Information

S1 Table Adjusted annual percent change in functional and activity limitations based on multivariate logistic model, 1998–2012—with additional controls (95% confidence intervals are in parentheses). (DOCX)

S2 Table Adjusted annual percent change in functional limitations and activity limitations based on logistics models with various controls, 1998–2012 –by Gender (95% confidence intervals are in parentheses). (DOCX)

S3 Table Adjusted annual percent change in functional limitations and activity limitations based on multivariate logistic regressions with various controls—including both community dwelling and nursing home population, 2000–2010 (95% confidence intervals are in parentheses). (DOCX)

S4 Table Projection of educational attainment for the population aged 55–69 (%; based on data from the 2012 Current Population Survey). (DOCX)

S5 Table Weighted unadjusted prevalence of functional limitations, activity limitations, and covariates for ages 55–69 –by education level. (DOCX)
