# Supplementary material for: Are Functional and Activity Limitations Becoming More Prevalent among 55 to 69-Year-Olds in the United States?
Source: PLoS One. 2016 Oct 26;11(10):e0164565. doi: 10.1371/journal.pone.0164565 (PMC5082687; doi:10.1371/journal.pone.0164565)
Supplement: S1 Table — (DOCX) [file pone.0164565.s001.docx]

S1 Table. Adjusted annual percent change in functional and activity limitations based on multivariate logistic model, 1998-2012 - **with additional controls**

(95% confidence intervals are in parentheses)

| Outcome |  | |
| --- | --- | --- |
| Functional limitations |  |  |
| Vision: Poor or legally blind | 0.09% | |
|  | (-1.04 | 1.17) |
|  |  |  |
| Hearing: Poor | 1.36% | |
|  | (-0.26 | 2.20) |
|  |  |  |
| Cognition: CIND or demented  (self-reports only) | -0.30% | |
|  | (-0.80 | 0.37) |
|  |  |  |
| Physical functioning: any of 9 limitations | -0.54% | |
|  | (-0.64 | -0.13) |
|  |  |  |
| Activity limitations |  |  |
| Any of 5 IADLs | 1.66% | |
|  | (0.52 | 1.84) |
|  |  |  |
| Any of 6 ADLs | -0.23% | |
|  | (-0.91 | 0.57) |
| Controls |  |  |
| Age, gender, proxy, mode | X | |
| Race/ethnicity, being foreign born, marital  status | X | |
