# Supplementary material for: Are Functional and Activity Limitations Becoming More Prevalent among 55 to 69-Year-Olds in the United States?
Source: PLoS One. 2016 Oct 26;11(10):e0164565. doi: 10.1371/journal.pone.0164565 (PMC5082687; doi:10.1371/journal.pone.0164565)
Supplement: S2 Table — (DOCX) [file pone.0164565.s002.docx]

S2 Table. Adjusted annual percent change in functional limitations and activity limitations based on logistics models with various controls, 1998-2012 – **by Gender**

(95% confidence intervals are in parentheses)

|  | Men | | | | | | | |  | Women | | | | | | | |
| --- | --- | --- | --- | --- | --- | --- | --- | --- | --- | --- | --- | --- | --- | --- | --- | --- | --- |
| Outcome | Model 1 | | Model 2 | | Model 3 | | Model 4 | |  | Model 1 | | Model 2 | | Model 3 | | Model 4 | |
| Functional limitations |  |  |  |  |  |  |  |  |  |  |  |  |  |  |  |  |  |
| Vision: Poor or legally blind | 0.09% | | 1.95% | | 0.54% | | -0.05% | |  | 0.30% | | 2.65% | | 0.59% | | -0.14% | |
|  | (-2.04 | 2.22) | (-0.08 | 3.97) | (-1.61 | 2.67) | (-2.19 | 2.09) |  | (-1.02 | 1.62) | (1.51 | 3.79) | (-0.73 | 1.90) | (-1.46 | 1.19) |
|  |  |  |  |  |  |  |  |  |  |  |  |  |  |  |  |  |  |
| Hearing: Poor | 0.83% | | 1.59% | | 1.17% | | 0.60% | |  | 1.04% | | 2.26% | | 1.16% | | 0.52% | |
|  | (-0.75 | 2.40) | (0.06 | 3.13) | (-0.40 | 2.74) | (-0.99 | 2.20) |  | (-0.90 | 2.99) | (0.33 | 4.20) | (-0.81 | 3.12) | (-1.44 | 2.48) |
|  |  |  |  |  |  |  |  |  |  |  |  |  |  |  |  |  |  |
| Cognition: CIND or demented (self-reports only) | 0.09% | | 2.34% | | 0.29% | | 0.02% | |  | 0.31% | | 3.23% | | 0.47% | | -0.01% | |
|  | (-0.81 | 0.98) | (1.57 | 3.10) | (-0.57 | 1.15) | (-0.89 | 0.94) |  | (-0.82 | 1.45) | (2.35 | 4.10) | (-0.67 | 1.61) | (-1.16 | 1.13) |
|  |  |  |  |  |  |  |  |  |  |  |  |  |  |  |  |  |  |
| Physical functioning: any of 9 limitations | -0.16% | | 0.27% | | 0.03% | | -0.52% | |  | -0.50% | | -0.02% | | -0.45% | | -0.84% | |
|  | (-0.54 | 0.23) | (-0.10 | 0.63) | (-0.34 | 0.40) | (-0.88 | -0.16) |  | (-0.79 | -0.21) | (-0.29 | 0.25) | (-0.74 | -0.15) | (-1.11 | -0.57) |
| Activity limitations |  |  |  |  |  |  |  |  |  |  |  |  |  |  |  |  |  |
| Any of 5 IADLs | 0.90% | | 2.06% | | 1.21% | | 0.46% | |  | 1.61% | | 3.19% | | 1.86% | | 0.82% | |
|  | (-0.13 | 1.94) | (1.09 | 3.04) | (0.22 | 2.20) | (-0.60 | 1.52) |  | (0.56 | 2.65) | (2.15 | 4.22) | (0.81 | 2.91) | (-0.19 | 1.84) |
|  |  |  |  |  |  |  |  |  |  |  |  |  |  |  |  |  |  |
| Any of 6 ADLs | 0.88% | | 1.95% | | 1.18% | | -0.01% | |  | -0.60% | | 0.87% | | -0.43% | | -1.53% | |
|  | (-0.21 | 1.97) | (0.94 | 2.94) | (0.12 | 2.24) | (-1.04 | 1.02) |  | (-1.50 | 0.30) | (0.02 | 1.73) | (-1.35 | 0.47) | (-2.40 | -0.67) |
| Controls |  |  |  |  |  |  |  |  |  |  |  |  |  |  |  |  |  |
| Age, gender, proxy, mode | x | | x | | x | | x | |  | x | | x | | x | | x | |
| Education |  |  | x | |  |  |  |  |  |  |  | x | |  |  |  |  |
| Smoking |  |  |  |  | x | |  |  |  |  |  |  |  | x | |  |  |
| Obesity |  |  |  |  |  |  | x | |  |  |  |  |  |  |  | x | |
