# Supplementary material for: Are Functional and Activity Limitations Becoming More Prevalent among 55 to 69-Year-Olds in the United States?
Source: PLoS One. 2016 Oct 26;11(10):e0164565. doi: 10.1371/journal.pone.0164565 (PMC5082687; doi:10.1371/journal.pone.0164565)
Supplement: S3 Table — (DOCX) [file pone.0164565.s003.docx]

S3 Table. Adjusted annual percent change in functional limitations and activity limitations based on multivariate logistic regressions with various controls - **including both community dwelling and nursing home population, 2000-2010** (95% confidence intervals are in parentheses)

| Outcome | Model 1 | | Model 2 | | Model 3 | | Model 4 | |
| --- | --- | --- | --- | --- | --- | --- | --- | --- |
| Functional limitations |  |  |  |  |  |  |  |  |
| Vision: Poor or legally blind (N=53,238) | -0.88% | | 1.57% | | -0.47% | | -1.23% | |
|  | (-1.79 | 0.54) | (0.00 | 2.24) | (-1.50 | 0.82) | (-2.04 | 0.28) |
|  |  |  |  |  |  |  |  |  |
| Hearing: Poor  (N=53,246) | 0.41% | | 1.47% | | 0.66% | | 0.00% | |
|  | (-1.07 | 1.66) | (-0.26 | 2.35) | (-0.93 | 1.86) | (-1.34 | 1.34) |
|  |  |  |  |  |  |  |  |  |
| Cognition: CIND or demented (self-reports only; N=49,896) | -0.29% | | 2.57% | | -0.12% | | -0.52% | |
|  | (-1.12 | 0.69) | (1.15 | 2.52) | (-0.98 | 0.81) | (-1.27 | 0.53) |
|  |  |  |  |  |  |  |  |  |
| Physical functioning: any of 9 limitations (N=52,312) | -0.35% | | 0.18% | | -0.23% | | -0.73% | |
|  | (-0.53 | 0.04) | (-0.13 | 0.39) | (-0.44 | 0.11) | (-0.77 | -0.26) |
|  |  |  |  |  |  |  |  |  |
| Activity limitations |  |  |  |  |  |  |  |  |
| Any of 5 IADLs  (N=50,323) | 0.95% | | 2.52% | | 1.26% | | 0.22% | |
|  | (-0.02 | 1.38) | (1.12 | 2.46) | (0.18 | 1.61) | (-0.52 | 0.83) |
|  |  |  |  |  |  |  |  |  |
| Any of 6 ADLs  (N=50,309) | 0.21% | | 1.66% | | 0.43% | | -0.79% | |
|  | (-0.56 | 0.86) | (0.50 | 1.88) | (-0.41 | 1.02) | (-1.22 | 0.10) |
| Controls |  |  |  |  |  |  |  |  |
| Age, gender, proxy, mode | x | | x | | x | | x | |
| Education |  |  | x | |  |  |  |  |
| Smoking |  |  |  |  | x | |  |  |
| Obesity |  |  |  |  |  |  | x | |
