# Supplementary material for: Are Functional and Activity Limitations Becoming More Prevalent among 55 to 69-Year-Olds in the United States?
Source: PLoS One. 2016 Oct 26;11(10):e0164565. doi: 10.1371/journal.pone.0164565 (PMC5082687; doi:10.1371/journal.pone.0164565)
Supplement: S4 Table — (DOCX) [file pone.0164565.s004.docx]

S4 Table. Projection of educational attainment for the population aged 55-69 (%; based on data from the 2012 Current Population Survey)

| 55-69 | < HS | HS | Some College | BA or more | Total |
| --- | --- | --- | --- | --- | --- |
| 2012 | 11.0 | 31.5 | 26.7 | 30.7 | 100.0 |
| 2013 | 10.8 | 31.6 | 26.9 | 30.7 | 100.0 |
| 2014 | 10.7 | 31.6 | 26.9 | 30.8 | 100.0 |
| 2015 | 10.5 | 31.7 | 27.1 | 30.7 | 100.0 |
| 2016 | 10.4 | 31.6 | 27.3 | 30.7 | 100.0 |
| 2017 | 10.4 | 31.6 | 27.3 | 30.7 | 100.0 |
| 2018 | 10.4 | 31.6 | 27.3 | 30.7 | 100.0 |
| 2019 | 10.5 | 31.6 | 27.2 | 30.6 | 100.0 |
| 2020 | 10.6 | 31.7 | 27.0 | 30.7 | 100.0 |
| 2021 | 10.7 | 31.8 | 26.8 | 30.6 | 100.0 |
| 2022 | 10.7 | 31.8 | 26.9 | 30.6 | 100.0 |
| 2023 | 10.8 | 31.7 | 26.8 | 30.7 | 100.0 |
| 2024 | 10.8 | 31.4 | 26.9 | 30.9 | 100.0 |
| 2025 | 10.9 | 31.1 | 27.0 | 31.1 | 100.0 |
| 2026 | 10.8 | 30.7 | 27.1 | 31.4 | 100.0 |
| 2027 | 10.9 | 30.4 | 26.9 | 31.7 | 100.0 |
| 2028 | 10.9 | 30.0 | 26.8 | 32.3 | 100.0 |
| 2029 | 11.0 | 29.5 | 27.0 | 32.5 | 100.0 |
| 2030 | 11.0 | 29.0 | 27.1 | 32.9 | 100.0 |
| 2031 | 11.0 | 28.6 | 27.3 | 33.2 | 100.0 |
| 2032 | 11.0 | 28.1 | 27.3 | 33.5 | 100.0 |
| 2033 | 11.1 | 27.8 | 27.2 | 33.9 | 100.0 |
| 2034 | 11.0 | 27.5 | 27.3 | 34.1 | 100.0 |
| 2035 | 11.0 | 27.2 | 27.5 | 34.4 | 100.0 |
| 2036 | 10.9 | 27.0 | 27.5 | 34.6 | 100.0 |
| 2037 | 11.1 | 26.6 | 27.6 | 34.8 | 100.0 |
