# Supplementary material for: Are Functional and Activity Limitations Becoming More Prevalent among 55 to 69-Year-Olds in the United States?
Source: PLoS One. 2016 Oct 26;11(10):e0164565. doi: 10.1371/journal.pone.0164565 (PMC5082687; doi:10.1371/journal.pone.0164565)
Supplement: S5 Table — (DOCX) [file pone.0164565.s005.docx]

S5 Table. Weighted unadjusted prevalence of functional limitations, activity limitations, and covariates for ages 55-69 – **by education level**

|  | 1998 | 2000 | 2002 | 2004 | 2006 | 2008 | 2010 | 2012 |
| --- | --- | --- | --- | --- | --- | --- | --- | --- |
| Vision: Poor or legally blind |  |  |  |  |  |  |  |  |
| < HS | 8.5 | 9.5 | 9.2 | 9.5 | 10.9 | 10.3 | 11.5 | 10.8 |
| HS | 3.2 | 3.4 | 3.0 | 3.9 | 3.3 | 3.1 | 3.8 | 5.4 |
| Some College | 2.1 | 2.2 | 2.0 | 2.2 | 2.5 | 2.9 | 3.1 | 3.7 |
| BA or more | 1.1 | 1.1 | 1.0 | 1.3 | 0.9 | 1.3 | 1.7 | 1.7 |
| Hearing: Poor |  |  |  |  |  |  |  |  |
| < HS | 5.3 | 5.1 | 5.7 | 5.1 | 4.8 | 4.9 | 4.8 | 4.9 |
| HS | 3.6 | 3.0 | 3.5 | 3.7 | 4.1 | 3.5 | 4.0 | 4.8 |
| Some College | 2.1 | 1.8 | 2.2 | 3.1 | 3.7 | 3.3 | 3.3 | 3.8 |
| BA or more | 1.5 | 1.4 | 1.5 | 1.6 | 2.1 | 2.0 | 1.6 | 2.0 |
| Cognition: CIND or demented (self-reports only) |  |  |  |  |  |  |  |  |
| < HS | 31.2 | 32.7 | 32.7 | 37.4 | 37.9 | 39.2 | 40.4 | 45.6 |
| HS | 8.6 | 10.6 | 9.7 | 10.7 | 11.9 | 13.3 | 13.4 | 14.0 |
| Some College | 4.4 | 4.9 | 5.7 | 6.1 | 6.8 | 7.1 | 8.3 | 9.1 |
| BA or more | 2.9 | 2.3 | 1.7 | 1.6 | 2.7 | 2.8 | 3.1 | 3.2 |
| Physical functioning: Any of 9 limitations |  |  |  |  |  |  |  |  |
| < HS | 69.5 | 70.8 | 72.3 | 73.0 | 74.1 | 75.1 | 73.2 | 72.6 |
| HS | 59.2 | 60.4 | 62.4 | 65.5 | 66.4 | 64.9 | 66.1 | 64.5 |
| Some College | 52.9 | 54.7 | 57.2 | 58.9 | 60.4 | 59.1 | 57.6 | 55.7 |
| BA or more | 42.8 | 41.2 | 42.8 | 42.7 | 43.0 | 42.5 | 42.2 | 40.9 |
| Any of 5 IADLs |  |  |  |  |  |  |  |  |
| < HS | 17.9 | 20.7 | 19.6 | 19.6 | 20.3 | 18.5 | 24.3 | 23.7 |
| HS | 7.9 | 8.0 | 9.0 | 9.3 | 10.7 | 10.6 | 12.9 | 13.2 |
| Some College | 5.9 | 6.9 | 7.7 | 9.0 | 9.3 | 9.6 | 10.1 | 10.9 |
| BA or more | 4.1 | 4.4 | 4.1 | 4.1 | 4.7 | 4.5 | 5.8 | 5.2 |
| Any of 6 ADLs |  |  |  |  |  |  |  |  |
| < HS | 20.5 | 21.9 | 24.0 | 23.4 | 25.8 | 23.5 | 28.4 | 26.1 |
| HS | 12.1 | 11.7 | 11.2 | 12.7 | 14.5 | 12.9 | 15.0 | 14.2 |
| Some College | 9.5 | 9.7 | 10.1 | 10.9 | 12.1 | 10.7 | 12.7 | 12.3 |
| BA or more | 5.1 | 6.7 | 5.6 | 6.2 | 6.7 | 6.3 | 7.1 | 6.1 |
